# Development of an iron overload HepG2 cell model using ferrous ammonium citrate

**DOI:** 10.1038/s41598-023-49072-7

**Published:** 2023-12-08

**Authors:** Usama Abbasi, Srinivas Abbina, Arshdeep Gill, Jayachandran N. Kizhakkedathu

**Affiliations:** 1 Department of Pathology and Laboratory Medicine, The University of British Columbia, Vancouver, BC Canada; 2 Centre for Blood Research, Life Sciences Institute, The University of British Columbia, Vancouver, BC Canada; 3 Department of Chemistry, The University of British Columbia, Vancouver, BC Canada; 4 The School of Biomedical Engineering, The University of British Columbia, Vancouver, BC Canada

**Keywords:** Biological techniques, Cell biology

## Abstract

Cell-based iron overload models provide tremendous utility for the investigations into the pathogenesis of different diseases as well as assessing efficacy of various therapeutic strategies. In the literature, establishing such models vary widely with regards to cell lines, iron source, iron treatment conditions and duration. Due to this diversity, researchers reported significant differences in the measured outcomes, either in cellular function or response to a stimulus. Herein, we report the process required to establish an iron overload HepG2 cell model to achieve a consistent and reproducible results such that the literature can strive towards a consensus. Iron loading in cells was achieved with 50 μM of iron every 24 h for 2 days, followed by an additional 24 h of maintenance in fresh media. We demonstrated that iron overloaded cells had significantly increased ROS generation, labile and total iron whilst having various cellular functions resemble cells without iron overload. The present report addresses key pitfalls with regards to the lack of consensus currently present in the literature.

## Introduction

Iron is an essential mineral for all eukaryotes; it serves as a prosthetic group for a variety of proteins involved in central cellular processes^1–3^. As such, vertebrates have evolved highly specialized mechanisms that tightly regulate iron uptake, transport and utilization, recycling, and storage. The liver plays a central role in coordinating iron homeostasis; all acquired iron enters the liver^4,5^, where it is either stored or utilized intracellularly, or mobilized for systemic demands^6^. At cellular level, iron trafficking in liver is mainly controlled by hepatocytes and liver resident macrophages, Kupffer cells^7^. Approximately, 30% of the total amount of iron in the body is stored in the liver^8,9^. A perturbance in iron homeostasis can result in iron overload either due to genetic disorders that give rise to hereditary hemochromatosis, or acquired as a result of another disease (including thalassemia, acquired anemia, myelodysplastic syndromes and dysmetabolic iron overload syndrome) that give rise to secondary hemochromatosis with excess liver iron^6,10,11^. Moreover, excess free iron is directly correlated to hepatic tissue injury, giving rise to hepatic insulin resistance^12,13^, fibrosis and cirrhosis^14,15^, and increased risk for hepatocellular carcinoma^16,17^.

Acquired iron, either transferrin-bound iron (TBI) or non-transferrin bound iron (NTBI) under pathological conditions, enters a transient pool of redox-active iron referred to as the labile iron pool (LIP)^18–21^. This metabolically active iron serves as a crossroads of metabolic pathways^20^. In diseases states, where there is elevated labile iron, toxicity ensues through the generation of reactive oxygen species (ROS) via the Haber–Weiss reaction, which imparts oxidative damage to lipids, proteins, and DNA^22–24^. Ultimately, these events disrupt vital metabolic functions leading to cell cycle arrest, apoptosis and necrosis^25,26^. Thus, it is important to have a reliable and reproducible iron overload model to study biology and evaluate therapeutics that influence iron homeostasis.

There are numerous investigations describing the effect of hepatic iron overload on various cellular functions in the context of different diseases including infection and inflammation, lipid metabolism, insulin resistance, liver diseases and iron uptake^27–40^. However, the in vitro iron overload models established to study such conditions are diverse with regards to (a) hepatic cell lines, (b) iron source and (c) iron treatment conditions. For example, Hirsh et al*.* eloquently highlight the differences in total iron concentrations in HepG2 and Hep3B cells in response to both transferrin-bound iron as well as non-transferrin bound iron^37^. This implies that concentration and source of iron is critical to understand how the cellular iron overload model will respond to iron loading. This may result in differences in cellular function and protein expression that could influence the downstream biology. In addition, various groups have used multiple sources of iron ranging from citrate^27,30,32,37,38,40–45^, nitriloacetate^29,36,44,46,47^, and sulfate^33,34,39,48,49^ iron complexes with concentrations ranging from as low as 1 μM to as high as 10 mM, and also the treatment time varies as short as 4 h to as long as 7 days^27,32–34,36–48,50^ (see Supplementary Table S1 for detailed information). While it is difficult to directly compare these models, the iron concentration in the media required to load HepG2 cells with sufficient cellular iron and induce cytotoxicity seem to be dependent on the type of iron source rather than the media concentration of iron itself. For instance, Zibert and co-workers recently developed an iron overload model using HepG2 cells by treating them with around 10 mM of iron citrate (Fe^3+^), iron sulfate (Fe^2+^), and iron chloride (Fe^3+^) for 4 h^51^. Although, cells were healthy up to 48 h, iron treatments for 4 h may not be a viable method for different studies. In another study, Sekine et al*.* monitored the cytocompatibility of Hep39b and HeLa cells for six days in an iron overload model, treated with different concentrations of Fe-NTA, ranging from 30, 100, 300, 500 and 1000 μM. Except for 30 μM, the rest of the treatments showed significant toxicity compared to non-treatment control^29^. As mentioned above, different iron sources have significant effect on iron loading. For instance, Barisani et al*.* used ferric ammonium citrate (~ 720 μM) to load HepG2 cells. In this model, cell toxicity was not observed up to 7 days^32^.

Consequently, the differences in iron source and treatment duration can introduce confound variables that may result in differences in measured outcomes impacting the comparison of these findings across studies in literature. This is best exemplified by Fang et al*.* and Petrak et al*.* who performed proteomic analysis of HepG2 cells loaded with different concentrations of ferrous sulfate. They discovered that the expression of a number of proteins was sensitive to the iron concentration used^33,34^. This underlines the importance of having a consistent and reproducible in vitro iron overload model to study biology and evaluate therapeutics. Herein, we report the development of an iron overload HepG2 cell model, using ferric ammonium citrate (FAC), with a particular focus on the generation of ROS, and iron status with regards to labile and total iron.

## Methods

### Materials

Pierce Radioimmunoprecipitation assay (RIPA) lysis buffer, PBS buffer, Eagle’s Minimum Essential Media (EMEM), Trypan blue, 2 mg/mL bovine serum albumin (BSA) standards, cell scrapers, and Pierce Coomassie (Bradford) protein assay kit were purchased from Thermo Fisher Scientific unless otherwise mentioned. Fetal bovine serum, tissue culture treated 6-well and 48-well cell culture plates, tissue culture treated T25 flasks, concentrated nitric acid, ammonium acetate, ferric ammonium citrate (FAC), ferene (3-(2-Pyridyl)-5,6-di(2-furyl)-1,2,4-triazine-5′,5′′-disulfonic acid disodium salt), calcein acetoxymethyl ester (Cal-AM) and sodium L-ascorbate were obtained from Sigma-Aldrich. 7-AAD solution was acquired from BioLegends. For cellular assay kits, both the DCFDA/H2DCFDA ROS generation kit (ab113851) and the JC-10 mitochondrial membrane potential assay kit (ab112134) were purchased from Abcam, while the MTT assay kit was purchased from ATCC (20-1010K).

### Cell culture

HepG2 cells, a hepatocarcinoma cell line (ATCC HB-8065), with passage numbers between 3 and 10 were used for all experiments. Cells were maintained in EMEM with media changed every 1–2 days. For all iron treatments, ferric ammonium citrate (FAC) was used as the source of iron (18 mol% iron as described by manufacture, obtained from Sigma-Aldrich). All concentrations were prepared with respect to iron in EMEM.

### Treatment of cells with ferric ammonium citrate

For iron loading conditions, HepG2 cells were seeded in 6 wells plates at a density of 400,000 cells per well and grown for 2 days. Cells were treated with 1 mL media containing either 0, 50, 100, 200, 500 or 1000 μM iron from FAC each day for either 1 or 2 days. After iron loading, cells were washed with 1 mL PBS thrice. Cell lysates and subsequent total iron analysis were performed, as outlined below.

For iron retention conditions, HepG2 cells were seeded in 6 well plates at a density of 740,000 cells per well and grown for 2 days. Cells were then iron loaded by treating with 1 mL of 200 μM iron in the media each day for 2 days. Then, cells were maintained for an additional 0, 1, 2 or 3 days in 1 mL of media without iron with the media replaced daily. Cells were washed with 1 mL PBS thrice. Cell lysates and subsequent total iron analysis were performed, as outlined below. In addition to this, iron overloading was achieved using the treatment of cells with FeSO_4_.7H_2_O and FeCl_3_.6H_2_O (Fig. S3, supplementary information), however, FAC generated more reliable results.

### Final protocol for establishing an iron overload HepG2 model

The final protocol for establishing an iron overload model in HepG2 was followed; cells were treated with iron-containing media each day for 2 days, followed by maintaining the cells in media without iron for 1 day.

### Iron overloading HepG2 cells

To assess changes in the iron overload model due to varying iron loading concentrations, cells were iron loaded with either 50, 100 or 200 μM of iron following the “Iron overloading HepG2 model” protocol. Prior to any analysis, cells were washed with PBS thrice.

For changes in total and labile iron, HepG2 cells were cultured at a density of 500,000 cells per T25 tissue culture flask. After following the iron overload model protocol, cell lysates were prepared and iron was quantified, as outlined below. For changes in ROS generation and calcein-based labile iron, HepG2 cells were cultured in 6 well plates at a density of 400,000 cells per well. Cells were stained and analyzed via flow cytometry, as described below. After being iron overloaded, HepG2 cells were also characterized for their cellular functions including metabolic activity (see MTT assay), mitochondrial membrane potential (see JC-10 assay), membrane integrity (see Trypan Blue assay), and cell viability (see 7-AAD assay). For the MTT and JC-10 assay, cells were seeded in a 48 well plate at a density of 50,000 cells per well while for the Trypan blue and the 7-AAD assay, cells were cultured in 6 well plates at a density of 400,000 cells per well.

### Iron overload HepG2 cell culture and treatment with iron chelators

HepG2 cells were iron loaded with 50 μM following the “Iron overloading HepG2 model” protocol. After iron loading, cells were washed twice with PBS and then treated with 15 μM of iron chelators deferoxamine (DFO) or deferiprone (DFP) or deferasirox (DFX) prepared in EMEM for 48 h. Cells were washed thrice with PBS and analyzed for ROS generation, changes in labile and total iron, and changes in transferrin receptor 1 expression—as outlined below. This experiment was done in triplicates. Non-iron loaded HepG2 cells were analyzed as negative control. Cells were seeded in 6 well plates at a density of 400,000 cells per well for each study.

### Cell lysate preparation and protein measurement

HepG2 cells were scraped and pelleted at 500 g for 5 min. Harvested cells were lysed in 300 μL Pierce RIPA buffer with sonication. Cell debris was pelleted, and the supernatants were quantitatively collected. Protein content was measured by Bradford assay. A standard curve was generated using BSA and sample concentrations were interpolated. Samples were kept at − 80 °C prior to any further analysis.

### Iron quantification

A modified ferene assay was used to quantify both labile and total iron from cell lysates, as described elsewhere^52^. Iron standards were prepared from FAC in 4% nitric acid, ranging from 0 to 1000 μM.

#### Labile iron measurement

Labile iron concentrations were determined from cell lysates prepared. 100 μL of cell lysates and 100 μL iron standards were transferred into clean Eppendorf tubes. To each, 100 μL ammonium acetate buffer (pH 4.5, 2.5 M) and 120 μL working solution (5 mM ferene and 10 mM ascorbic acid) were added and left overnight. This mixture was spun at 21,000 g for 10 min to pellet any insoluble salts and debris. Absorbance was read at 595 nm on a SpectraMax 190 Microplate Reader from Molecular Devices. Labile iron concentrations were interpolated from a standard curve generated by using iron standards.

#### Total iron measurements

Total iron concentrations were determined from cell lysates by first digesting them with concentrated nitric acid maintained at 100 °C to 120 °C followed by resuspension of the dried acid-digested samples in 200 μL of 4% nitric acid. Iron standards (200 μL) were transferred into clean Eppendorf tubes. To each, 200 μL ammonium acetate buffer (pH 4.5, 2.5 M) and 240 μL working solution (5 mM ferene and 1 M ascorbic acid) were added. This mixture was vortexed and left overnight. Absorbance was measured at 595 nm and sample concentrations were interpolated from the standard curves generated. Total iron measurements were also performed by inductively coupled plasma mass spectrometry (ICP-MS) (Fig. S4, supplementary information)^52^.

### ROS measurements

Cellular ROS generation measured using a DCFDA/H_2_DCFDA kit (Abcam 113851) on Beckman Coulter CytoFLEX Flow Cytometer. In short, DCFDA, a fluorogenic cell permeable dye, undergoes deacetylation by cellular esterases to a non-fluorescent dye. Cellular ROS oxidize this molecule into DCF which is highly fluorescent, detected in the FITC channel. Manufacturer’s protocol was followed. In brief, after washing cells with PBS thrice, cells were stained with 5 μM DCFDA in EMEM and incubated for 20 min at 37 °C. Then, cells were washed with PBS, trypsinized and pelleted. At least 10,000 cells were analyzed via flow cytometry and DCF was measured by the 488 nm laser and the FITC emission filter (530/20 nm).

### Calcein-based labile iron measurements

Calcein acetoxymethyl ester (Cal-AM) was used to measure changes in the intracellular labile iron, described elsewhere^53^. Cal-AM is a non-fluorescent dye that readily permeates the cell membrane and is cleaved into calcein, which is fluorescent^54,55^. Calcein then binds to iron stoichiometrically which quenches its green fluorescence^54,55^. In short, cells were washed, trypsinized and pelleted at 500 g for 5 min. Cells were resuspended in PBS with 0.2 μM of Cal-AM and incubated for 20 min at room temperature. At least 10,000 cells were analyzed via flow cytometry and calcein fluorescence was measured by the 488 nm laser and the FITC emission filter (530/20 nm).

### Metabolic activity by MTT assay

The MTT (3-(4,5-dimethylthiazolyl-2)-2,5-diphenyltetrazolium bromide) assay (ATCC 20-1010 K) was performed according to the manufacturer’s protocol to investigate changes in metabolic activity. In brief, after iron loading, cells were treated with MTT for 2 h followed by detergent-induced lysis for 4 h. Absorbance was measured at 570 nm on SpectraMax 190 Microplate Reader from Molecular Devices. Cell viability was determined; (mean_570 nm_ treated cells/mean_570 nm_ untreated cells) × 100%.

### Mitochondrial membrane potential by JC-10 assay

The JC-10 mitochondrial membrane potential assay kit (abcam 112134) was performed, in accordance with the manufacturer’s protocol, to investigate changes in mitochondrial membrane potential. JC-10 localizes in the mitochondria and changes fluorescent emission from green to orange as membrane potential increases; its monomeric form, which emits at 520 nm, forms aggregates, which emits at 590 nm, as the mitochondria becomes more polarized. In brief, iron overload HepG2 cells were treated with JC-10 for 30 min at 37 °C and fluorescence was measured at 490/525 and 540/590 (excitation/emission nm) on a SpectraMax 190 Microplate Reader from Molecular Devices. Mitochondrial membrane potential was determined as follows ((ratio of 520 nm/590 nm in treated cells)/(ratio of 520 nm/590 nm in control cells)) × 100%.

### Membrane integrity by Trypan blue exclusion assay

Membrane integrity was investigated using the trypan blue exclusion method, as described elsewhere^56^. In brief, iron overloaded cells were scraped and pelleted, then resuspended in media with trypan blue (HyClone Trypan Blue Stain—Fisher). Live and dead cells were counted using a hemocytometer and percentages were reported.

### Cell viability by 7-amino-actinomycin D (7-AAD) assay

Cell viability was determined with a cell impermeable 7-AAD (7-amino-actinomycin D) solution (BioLegend) that fluoresces upon binding to DNA. In other words, 7-AAD fluorescence is indicative of membrane damage. Manufacturer’s protocol was followed. In short, iron overload cells were scraped and pelleted, then incubated with 5 μL of stock (50 μg/mL) per sample for 15 min at room temperature. Cells were then analyzed by flow cytometry; at least 10,000 cells were analyzed, and dead cells were gated in the APC channel (660/10 nm). Cell viability was reported as percentage of live cells (i.e., 7-AAD negative cells).

### Western blot analysis

Iron overload HepG2 cells were scraped and lysed; the corresponding protein concentrations were quantified as described above. Transferrin receptor 1 expression was investigated through western blots, as described elsewhere^57–60^. Proteins were separated in a 10% sodium dodecyl sulfate polyacrylamide gel and transferred to a nitrocellulose membrane. The membrane was blocked in 10% skim milk and incubated with both a monoclonal anti-transferrin receptor 1 (TfR1) antibody (H68.4—Thermofischer, 100 kDa), asialoglycoprotein receptor (ASGPR, 42 kDa) and GAPDH (Rabbit mAb, 37 kDa, Cell Signalling Technology,) at 3 μg/mL overnight. Primary antibodies were fluorescently tagged through fluorescently labelled secondary antibodies; donkey anti-mouse with an infrared dye 700 (LI-COR) was incubated at 1:10,000 for 4 h. The nitrocellulose membranes were imaged using LI-COR with resolution set at 169 μm, medium quality, 700 nm channel at intensity of 5, and scan area large enough to cover the membrane. The western blot was analyzed using LI-COR’s Odyssey Application Software 3.0. Data was first normalized to the housekeeping protein GAPDH, and then represented relative to the control cells; (ratio of TfR1:GAPDH in treated cells)/(ratio of TfR1:GAPDH in control cells).

### Flow cytometry analysis of surface proteins

Following iron loading protocol (Section: Iron overloading HepG2 cells). HepG2 cells were detached from 6-well plate using 1 ml of Trypsin–EDTA (Gibco 25200-072). Cells were incubated in trypsin for 10 min at 37 °C. EMEM media (4 mL, ATCC 30-2003) supplemented with 10% FBS was added to inactivate trypsin. Cells were centrifuged at 300 g for 5 min and the supernatant media was decanted. The cells were resuspended in 5 mL cold PBS, again centrifuged at 300 g for 5 min and PBS was decanted. The remaining cell pellet was resuspended in 300 µL of cold PBS. A 10 µL aliquot of cell suspension was set aside on ice for obtaining a cell count via a cell counter (Denovix CellDrop FL) with a trypsin assay. The remaining 290 µL of cell suspension was stained with FVD live/dead stain (Invitrogen L34973) at 4 °C for 20 min on a shaker. Cold PBS (1 mL) was then added, and cells were centrifuged for 5 min at 300 g. PBS was then aspirated out leaving the cell pellet behind. 4% PFA PBS solution (1 mL) of was then used to resuspend the cell pellet and fix the cells on ice for 15 min on a shaker. Meanwhile the previous 10 µL aliquot was used to count the cells. The fixed cells were then centrifuged for 6 min at 400 g, and the supernatant was aspirated. To the suspension, 1 mL PBS was added and again centrifuged for 6 min at 400 g, and the supernatant removed. Casein blocking buffer (1 mL, Sigma Aldrich 1003281044) was added to resuspend pellet and then centrifuged again for 6 min at 400 g. Cells were then resuspended in casein blocking buffer to give a concentration of 4 × 10^6^ live cells/ml (calculated from trypsin assay). Assay showed viability for controls at 94% and 87% for iron overloaded cells). 40 µL of the cell suspension (160,000 live cells) was used to incubate with 10 µL of a 1:4 dilution of both primary antibodies (anti-ferroportin polyclonal antibody from Invitrogen, pa5-143047; anti-GAPDH polyclonal antibody from Proteintech, 10494-1-ap) in PBS for 1 h on a shaker at room temperature. These dilutions were determined via antibody titration to find concentration of primary antibodies such that binding equilibrium was reached (titrations curve flattened out, Supplementary Fig. S7). After this, 1 mL of PBS was added and cells were centrifuged for 400 g at 6 min, supernatant aspirated, leaving 100 µL behind. Cells were then mixed with 30 µL of a PBS solution of both secondary antibodies (Anti-Goat Dylight 755 antibody at a 1:132 dilution of stock from Invitrogen, sa5-10091; Anti-Rabbit Alexa Fluor 488 at a 1:330 dilution of stock from Invitrogen, A21206) for 40 min at room temperature on a shaker. PBS (1 mL) was added, and cells were centrifuged again for 6 min at 400 g. Supernatant was aspirated leaving behind 100 µL. Cell pellet was then resuspended in 300 µL of PBS. Samples were then run on Flow cytometer (Beckman Coulter Cytoflex).

## Results

### Establishing an iron overload HepG2 model

**Figure 1:**
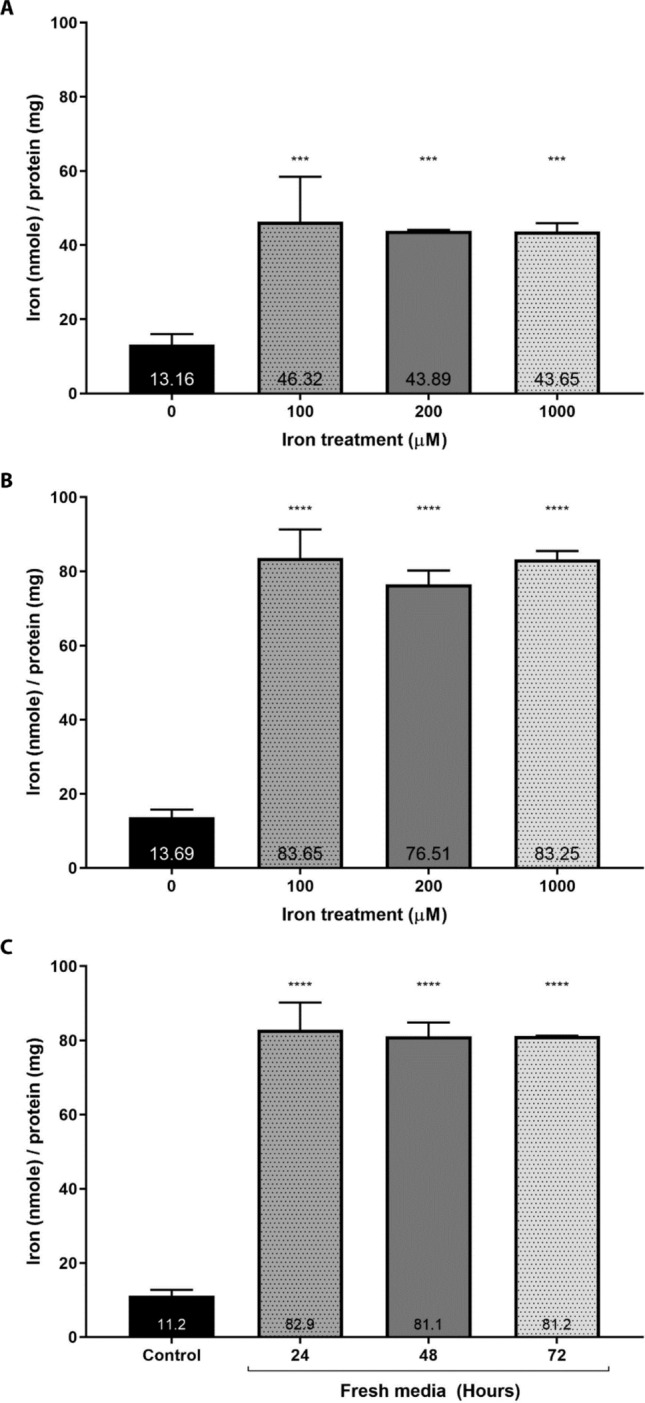
The effect of iron concentration and treatment time on establishing an iron overload HepG2 cell model. (**A** & **B**) HepG2 cells were treated with varying concentrations of iron (100 to 1000 μM) in the form of FAC either once in 24 h (**A**) or twice in 48 h (**B**). (**C**) HepG2 cells were first treated with 200 μM every 24 h for 48 h. Then, these cells were maintained in fresh media without iron for up to 72 h with media being changed every 24 h. Total iron was analyzed using* u*-ferene assay^52^. Error bars show standard deviations for N = 3 independent replicates. One-way ANOVA with Dunnett’s correction method were performed to compare differences against the control or non-iron overloaded cells. One-way ANOVA with Sidak’s correction method was performed to compare the iron loading with 24 h or 48-h treatment. ****Represents *p* < 0.0001 and ***represents *p* < 0.0010. All statistical analyses were performed using GraphPad Prism.

A protocol to establish an iron overload model for hepatocytes was developed using HepG2 cells and FAC as the source of non-transferrin bound iron (NTBI). First, the experimental conditions required to iron load HepG2 cells were explored (Fig. 1A,B). Cells were treated with varying concentrations of FAC for every 24 h for either 24 h or 48 h resulting in significantly higher total cellular iron concentrations when compared to non-iron treated cells. 24-h iron treatment resulted in 46.32 nmole iron/mg of protein (*p* = 0.0005), 43.89 nmole iron/mg of protein, (*p* = 0.0009), and 43.65 nmole iron/mg of protein (*p* = 0.0009) respectively for 100, 200, and 1000 μM FAC treatment. 48-h iron treatment further increased the cellular iron concentration in the range 76 to 84 nmole iron/mg of protein for all the treatment conditions and the values were significantly different from the control non-iron treated cells (*p* < 0.0001). Our data clearly show that cellular iron loading was dependent on the duration of the treatment rather than iron loading concentration. When comparing the different iron loading concentrations at either 24 h or 48 h, no significant difference is observed between 100, 200, or 1000 μM iron containing media. However, cells treated with 100, 200, and 1000 μM for 48 h yielded significantly higher total iron concentrations than the cells treated for 24 h (*p* < 0.0001, *p* < 0.0001, and *p* < 0.0001, respectively).

The duration of iron retention was investigated next (Fig. 1C). HepG2 cells were loaded with 200 μM every 24 h for 48 h. Then, these cells were maintained in fresh media for up to 3 days with media being replaced every day. Total iron concentrations did not change even up to 72-h post-loading in fresh media. Taken together, to establish a stable iron overload HepG2 cell model, cells were loaded with FAC every 24 h for 48 h followed by 24 h of maintenance in fresh media.

### The effect of iron loading concentrations on cellular iron responses

**Figure 2:**
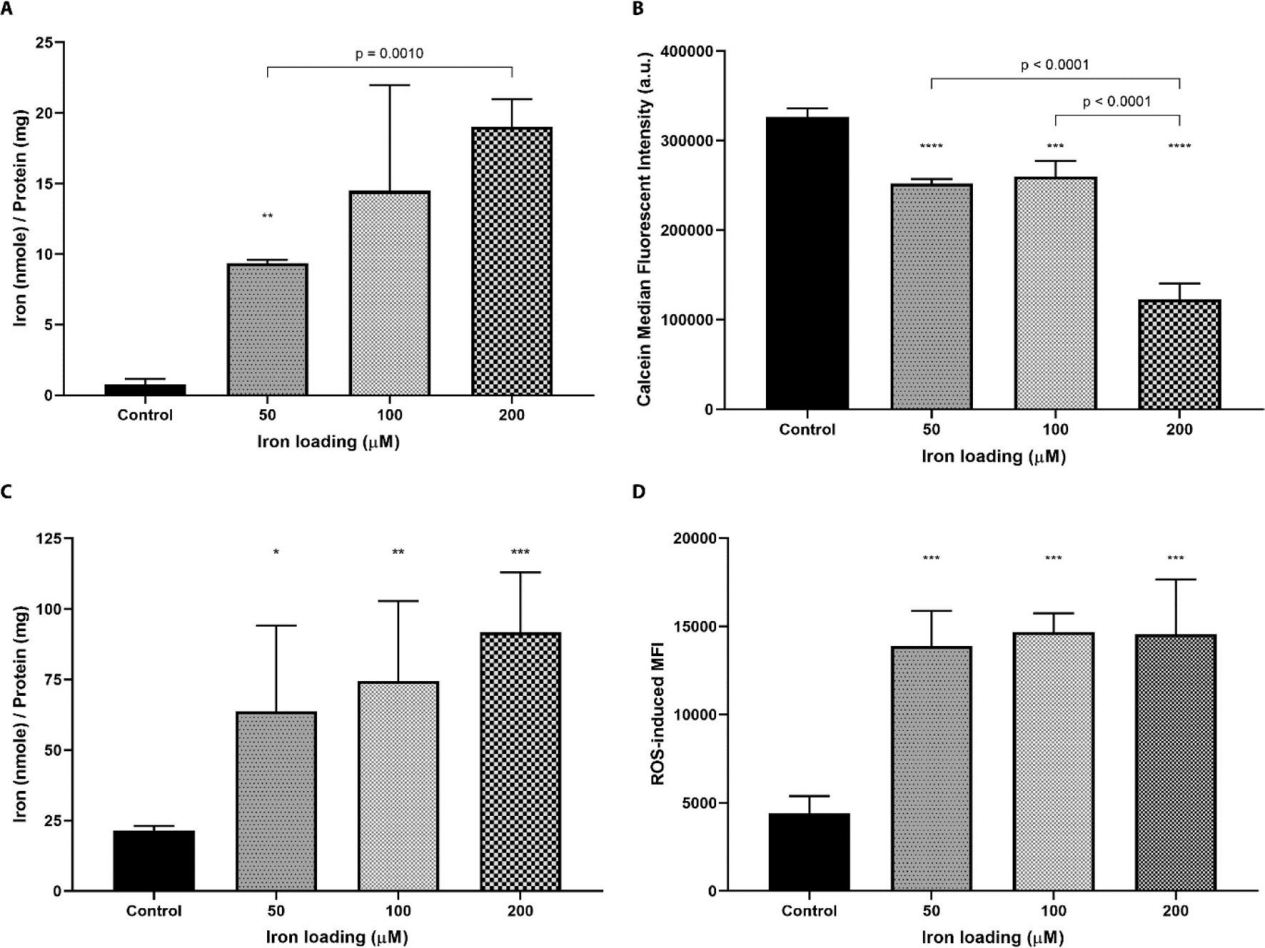
The effect of varying iron loading concentrations (50 to 200 μM) on labile iron, total iron and the generation of reactive oxidative species (ROS) in HepG2 cells. (**A**) Labile iron was quantified using the *u*-ferene assay. One-way ANOVA with Sidak’s correction method was used to compare iron-loaded cells with control cells. (**B**) Changes in the intracellular labile iron pool were monitored via fluorescence. Calcein fluorescence is quenched in the presence of iron. One-way ANOVA with either Sidak’s or Dunnett’s correction method was used to compare iron loaded cells with control cells. (**C**) Total iron was quantified using the *u*-ferene assay. One-way ANOVA with Dunnett’s correction method was used to compare iron loaded cells with control cells. (**D**) ROS generation was measured using a DCFDA/DCF ROS kit in at least 10,000 HepG2 cells via flow cytometry. One-way ANOVA with Dunnett’s correction method was used to compare iron loaded cells with control cells. Error bars show standard deviations for N = 3 independent replicates. Statistical analyses were performed using GraphPad Prism. ****Represent *p* < 0.0001, ***represents *p* < 0.0010, **represents *p* < 0.0100 and *represents *p* < 0.05.

**Figure 3:**
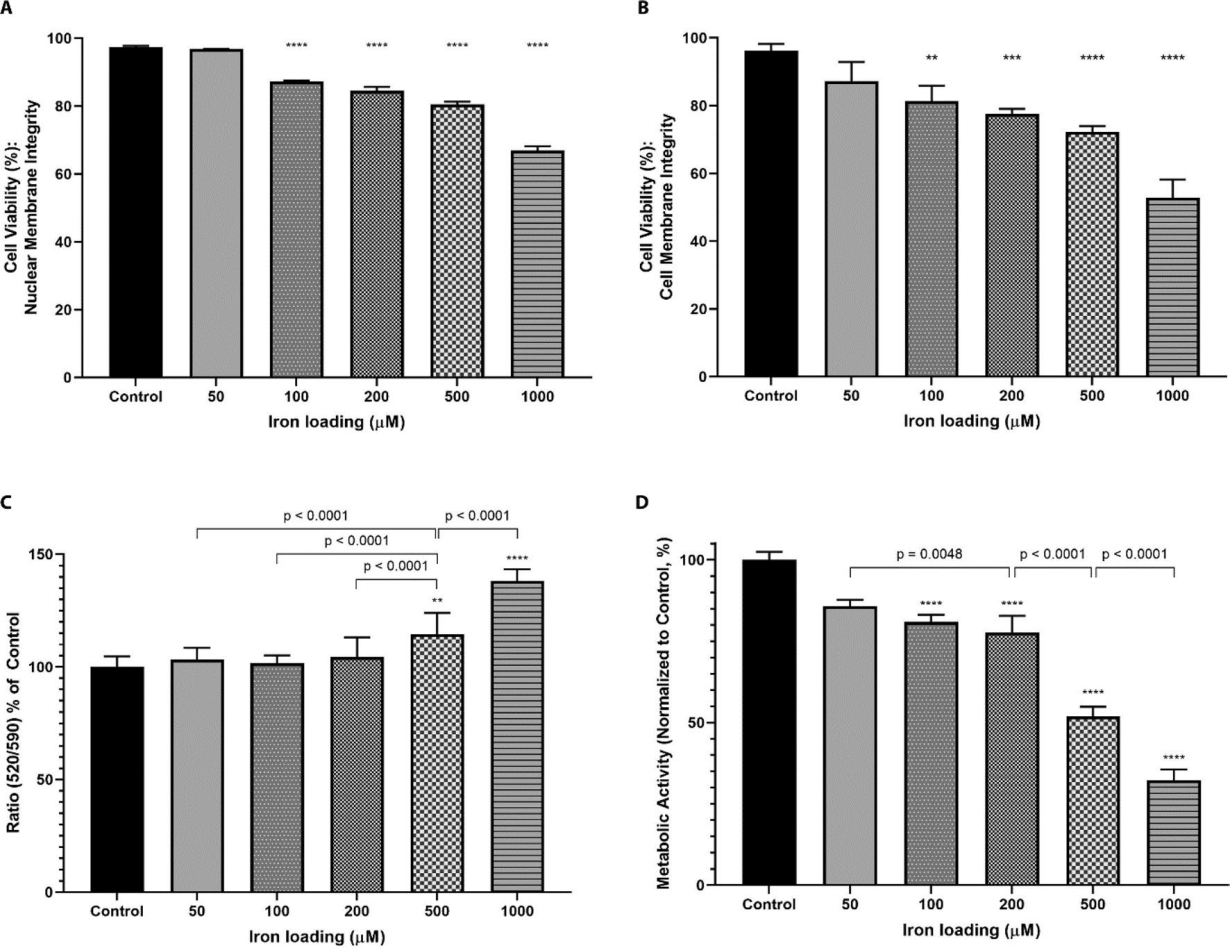
Characterization of cellular functions of HepG2 cells loaded with varying iron concentrations (50 to 1000 μM). (**A**) Cell viability was measured using a membrane impermeable 7-aminoactinomycin D (7-AAD) via a flow cytometry, analyzing at least 10,000 cells. 7-AAD binds to DNA and the fluorescence is detected in PC5.5 channel (710/50 nm). One-way ANOVA with Dunnett’s correction method were performed to compare iron loaded cells with control cells. (**B**) Cell membrane integrity was measured using the Trypan blue (TB) exclusion assay. This dye is membrane impermeable. Cells with or without TB uptake were counted using a hemocytometer. One-way ANOVA with Dunnett’s correction method were performed to compare iron-loaded cells with control cells. (**C**) Mitochondrial membrane potential was measured using the JC-10 assay kit using a plate reader. The ratio of fluorescence emitted at 590 nm and 540 nm were normalized to control cells. One-way ANOVA with Dunnett’s correction method were performed to compare iron loaded cells with control cells. Similarly, one-way ANOVA with Dunnett’s correction method were performed to compare iron loaded cells with cells loaded with 500 μM. (**D**) Metabolic activity was measured using the MTT assay using a plate reader. One-way ANOVA with Dunnett’s correction method were performed to compare iron loaded cells with control cells. One way ANOVA with Tukey’s correction was also performed to compare different iron loaded cells with each other. Error bars show standard deviations for 3–6 independent replicates. All statistical analyses were performed using GraphPad Prism. ****Represents *p* < 0.0001, ***represents *p* < 0.0010, and **represents *p* < 0.0100.

Iron overload HepG2 cells were established using different iron loading concentrations (50, 100, and 200 μM) using the optimized protocol to investigate the changes in cellular response with respect to iron loading (Fig. 2) and cellular functions (Fig. 3). Key parameters examined in cellular iron responses including labile iron concentration (Fig. 2A,B), total iron concentration (Fig. 2C) and ROS generation (Fig. 2D). Iron loaded cells showed a concentration-dependent increase in the labile iron pool (LIP). There is a significant increase in the LIP quantified by the *u*-ferene assay when comparing non-loaded cells to cells loaded with 50 μM iron or cells loaded with 50 μM iron compared to 200 μM iron (*p* = 0.0066 and *p* = 0.0010, respectively). The dose-dependent increase in the LIP is further corroborated by the calcein-based fluorescent assay; calcein fluorescence reduces as it binds to labile or redox active iron. The significant reduction in calcein-based fluorescence between control cells and 50 μM iron overloaded cells and between 50 and 200 μM iron overloaded cells (*p* = 0.0001 and *p* < 0.0001, respectively) is suggestive of increasing labile iron concentrations.

Similar to iron loading investigations (Fig. 1A), there is an increase in total iron concentration (Fig. 2C) when cells are treated with 50, 100, and 200 μM of iron-containing media compared to controls (*p* = 0.0165, *p* = 0.0030 and *p* = 0.0006, respectively). Further, there is no appreciable differences in total iron loading amongst iron treated cells with different concentrations highlighting the reproducibility of establishing a HepG2-based iron overload cell model.

Interestingly, the concentration-dependent trend in labile iron concentrations is not observed for ROS generation (Fig. 2D). There was a significant increase in ROS mediated median fluorescence intensity when comparing non-iron loaded cells to iron loaded cells (*p* = 0.0010, *p* = 0.0006 and *p* = 0.0006 for cells loaded with 50, 100 and 200 μM iron, respectively). However, there were no significant differences when comparing iron overloaded cells at any loading concentrations tested. Despite increasing labile iron concentrations, ROS generation does not increase with respect to increasing iron loading concentrations.

### Characterization of cellular functions in response to varying iron loading concentration

Cellular functions were characterized in iron overloaded HepG2 cells. Cell viability was measured as a function of either nuclear or plasma membrane integrity (Fig. 3A,B). In both cell viability assays, HepG2 cells loaded with 50 μM iron showed no significant differences when compared to control (non-iron loaded cells). Any iron loading concentrations above 100 μM yielded a significant increase in either nuclear or plasma membrane permeability when compared to controls (*p* < 0.0001 for all comparisons). Mitochondrial membrane potential was measured through the JC-10 assay, which takes advantage of the different fluorescent emissions when cells become apoptotic (Fig. 3C). HepG2 cells loaded with iron at 500 μM or 1000 μM had perturbed the mitochondrial membrane potential significantly when compared to the control (*p* < 0.0001 for both). No significant alterations in cells loaded with 50, 100 or 200 μM iron were observed. Metabolic activity was observed through the MTT assay which measures the rate of MTT conversion to formazan salt by intracellular enzymes, including dehydrogenase enzymes (Fig. 3D). All iron overloaded HepG2 cells yielded significantly lower metabolic activities when compared to the control (*p* < 0.0001 for all comparisons). Taken together, HepG2 cells loaded with 50 μM were similar to control cells with respect to nuclear membrane integrity, plasma membrane integrity and mitochondrial membrane potential. As iron loading concentrations increased, particularly at 200 μM or higher, cellular functions were significantly different to iron overload cells loaded with lower iron concentrations.

### Application of the iron overload model to investigate iron chelation in HepG2 cells

**Figure 4:**
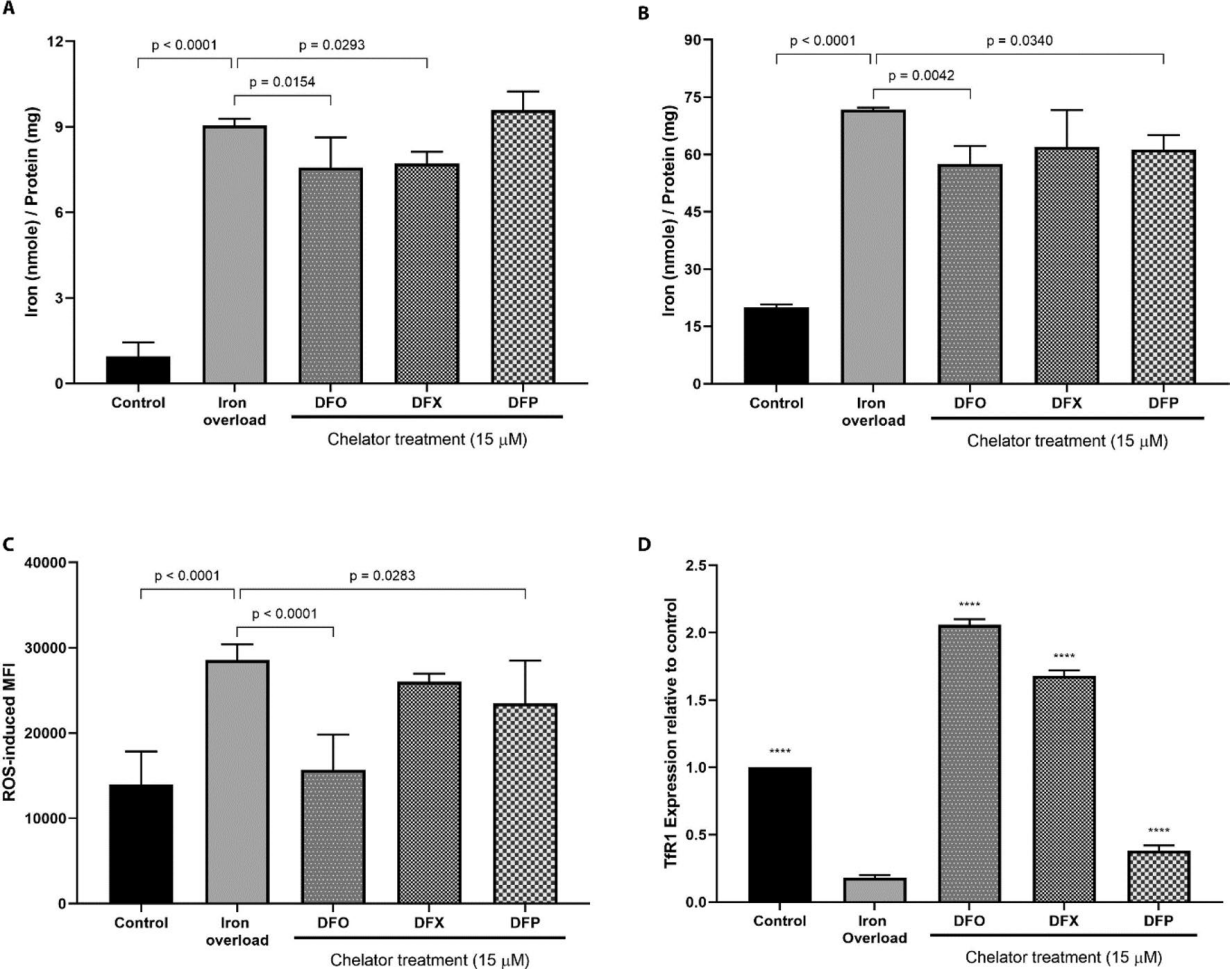
Effect of iron chelation in iron overload HepG2 cells. Labile iron (**A**) and total iron (**B**) were quantified using the *u-*ferene assay. (**C**) ROS generation was measured using a DCFDA/DCF ROS kit in at least 10,000 cells via flow cytometry. (**D**) Relative expression of TfR1, normalized to control cells, were measured using western blot and quantified using LI-COR’s Odyssey Software Application. HepG2 cells were loaded with 50 μM following the established protocol. Error bars show standard deviations for N = 3 independent replicates. One-way ANOVA with Dunnett’s correction method were performed to compare iron overloaded cells with the either control or chelator treated cells. Statistical analyses were performed using GraphPad Prism. ****Represents *p* < 0.0001.

HepG2 cells loaded with 50 μM iron were treated with 15 μM DFO, DFX and DFP for 48 h to investigate changes in iron concentrations, ROS, and protein (TfR1, ASGPR and GAPDH) expression. The purpose of this experiment was to test whether the model could be used for evaluating therapeutics and how it impacts the cellular physiology. No changes were observed in cell viability with this treatment (Supplementary Fig. S1). All chelators showed significant reduction in iron concentrations after the treatment. DFO treated cells had a significant reduction in both LIP (Fig. 4A) and total iron concentrations (*p* = 0.0154 and *p* = 0.0042, respectively (Fig. 4B), DFX treated cells only had a significant reduction in LIP concentrations (*p* = 0.0293), and DFP treated cells only had a significant reduction in total iron concentrations (*p* = 0.0340) (Fig. 4A,B). Further, DFO and DFP reduced intracellular ROS generation when compared to untreated iron overload cells (*p* < 0.0001 and *p* = 0.0283, respectively). Interestingly, DFO treated cells showed ROS levels comparable to the non-iron loaded control cells. DFX showed no changes in ROS (Fig. 4C).

Transferrin receptor 1 (TfR1) expression was measured as cellular response to iron overload. Under iron overload conditions, TfR1 expression decreased when compared to control and iron overload HepG2 cells (*p* < 0.0001). Upon treatment with iron chelators significantly increased TfR1 expression when compared to iron overload cells (*p* < 0.0001). Interestingly, DFO and DFX treatment resulted in expression levels greater than non-iron loaded control cells (Fig. 4D). We did not observe any major changes in the expression of ASGPR and GAPDH before and after treatment (Supplementary Fig. S5).

## Discussion

Liver iron overload where excess free iron results in hepatic toxicity and damage giving rise to a variety of disorders including hepatic insulin resistance^12,13^, fibrosis and cirrhosis^14,15^, and increased risk for hepatocellular carcinoma^16,17^. Therefore, in vitro hepatic iron overload models provide an invaluable tool to better understand pathogenesis whilst enabling the progression of treatment development. Such in vitro models exists, yet their utility is undermined by the extreme diversity in these studies, namely; (1) different hepatic cell lines, such has HepG2 versus Hep3B respond differently to NTBI, (2) various sources of NTBI are used, most commonly including citrate^27,30,32,37,38,40–45^, nitriloacetate^29,36,44,46,47^, and sulfate^33,34,39,48,49^ complexes, as well as other preparations^34,44,50,61^, and (3) iron treatment vary greatly with vastly different concentrations and durations, ranging from as low as 1 μM to as high as 2 mM for as short as 6 h to as long as 7 days^27,32–34,36–48,50^. A lack of consensus within the field can bias outcomes of interest. This is potentially highlighted in a proteomic study that investigated protein alterations in HepG2 cells loaded with two different iron concentrations; HepG2 cells treated with 10 μM resulted in 25 proteins being up-regulated and 5 proteins being down-regulated whereas HepG2 cells treated with 1000 μM resulted in 19 protein being up-regulated and 8 protein being down-regulated, with two down-regulated proteins at 10 μM becoming up-regulated at 1000 μM^33,34^. This motivated us to investigate this and establish a consistent iron overload in vitro model to enable reproducible findings.

Since iron treatment conditions vary greatly, HepG2 cells were first screened for changes in total iron with respect to concentration and duration of iron loading. Total iron in cells increased with the time of incubation rather than iron loading concentrations (Fig. 1); total iron concentrations were significantly more elevated in cells treated for 48 h compared to 24 h at the same concentrations, however, there were no significant differences between cells treated with different concentrations for a given time. Total iron concentrations in loaded HepG2 cells were in the range of 75 to 85 nmol per mg protein (3.5 to 5.0 μg iron per mg protein) after 48 h incubation, which is in agreement with earlier reports^37,45,62^. This has been validated through ICP-MS, the gold standard approach for elemental analysis in our earlier report (see Fig. S2, supporting information)^52^. Taken together, 48 h treatment periods is sufficient to induce iron overload, which is maintained for at least 3 days providing ample time to conduct further studies.

Next, we investigated the impact of iron loading concentrations using the established time stamps—load every 24 h for 48 h, followed by 24 h of fresh media. Intracellularly, a small fraction of redox labile iron is maintained in dynamic equilibrium such that it accounts for ~ 3–5% of the total iron^63^. This is observed using the *u*-ferene assay in control cells with 3.7% LIP (0.8 nmol of labile iron/mg of protein out of the 21.5 nmol of total iron/mg of protein). As shown in Fig. 1, HepG2 cells loaded with iron, irrespective of iron loading concentrations, showed no significant differences when comparing their total iron, highlighting the reproducibility of this iron overload model. Iron overloading using FAC gave consistent data in comparison to other iron sources such as FeCl_3_ and FeSO_4_ (Fig. S3, supporting information).

Moreover, similar to hepatic iron overload diseases, iron overloaded HepG2 cells demonstrate significantly elevated labile iron concentrations as iron loading concentrations increased. The labile iron concentration, using the *u*-ferene assay, was significantly increased when comparing control cells to iron overload HepG2 cells. This pattern was further corroborated with the calcein assay^46,48^. Elevated labile iron catalyzes the generation of ROS through the Haber–Weiss reaction, which imparts oxidative damage to lipids, proteins and DNA^22–24,64,65^. Intracellular ROS levels were measured and a significant increase was observed for iron overloaded cells. This increase in ROS has been documented in earlier reports^29,32,33,39,46^. Similar to total iron concentrations, ROS generation was not significantly different between cells treated with different iron loading concentrations. Huang et al*.*^39^ reports a linear correlation between lipid peroxidation and total iron concentrations, which might rationalize our findings. While it is also possible that HepG2 cells have internal mechanisms to handle and maintain such high ROS levels, further work would need to validate this. In addition, iron overload cells treated with hydrogen peroxide further increased ROS generation (*p* = 0.0024) (Supplementary Fig. S2) demonstrating that the cells are susceptible to further ROS generation and that ROS generation is still within the assay’s limit of detection. Taken together, using the established time stamps to develop an iron overload with different iron loading concentrations (50, 100 and 200 μM) only labile iron concentrations significantly changed.

Next, toxicity indicators were measured as a function of iron loading concentrations, lower iron loading concentrations better resembled non-iron loaded control cells in the measured cellular functions. As iron loading concentrations increased, iron overloaded HepG2 cells showed exacerbated decline in these parameters—as exemplified by HepG2 cells loaded with 1000 μM. Of importance, cytotoxicity indices vary greatly within literature. Abalea et al*.* report elevated LDH release in HepG2 cells treated with iron at 10 and 100 μM for 24 h whilst Fang et al*.* report unchanged HepG2 cell proliferation for concentrations as high as 1000 μM treated for up to 5 days^34,46^. It is important to distinguish that both Abalea et al*.* and Fang et al*.* used nitriloacetate complexed iron. Different NTBI sources yield different kinetic parameters with regards to NTBI uptake^44^. Previous publications, using FAC to treat HepG2 cells, also report varying toxicities. Parkes et al.^44^ report that nuclear membrane integrity changes at concentrations higher than 200 μM while LDH levels remain unchanged at concentrations as high as 1.4 mM. In addition, Popovic et al.^45^ report that metabolic activity, measured by MTT, decreases below 80% of control only at concentrations exceeding 2 mM. Given this variability and that many reports aim to investigate the treatment of iron overload or the reversal of ROS-mediated damage, it is essential that cytotoxic indices are thoroughly documented and account for different cellular parameters. Adopting this practice will empower reproducibility within the field. Our data showed that iron loading concentrations of 50 μM yield an iron overload model closest to control cells and that any concentration above 200 μM iron shows significant alterations to cellular functions.

We further performed a flow cytometry-based analysis to assess the cell surface protein expressions of GAPDH and ferroprotein. Our data shows no significant change in the levels of GAPDH and ferroportin post iron overload in our model (Supplementary Fig. S6) in contrary to the literature^66,67^. The explanation that we can postulate for this result is that our model is done in the HepG2 cell line (a liver cancer cell line) in vitro while literature studies were done in other cell lines such as macrophages and ex-vivo experiments on hepatocytes isolated from iron overloaded rats. While these studies show evidence for GAPDH increase in some cells upon iron loading, other cells show the opposite, suggesting inconsistency. Our explanation for no change in GAPDH and ferroportin expression is that since the liver is a main store of iron in the human body^9^. It is possible that the HEPG2 cells are able to handle the overload of iron in our model via storage rather than needing to necessarily export it.

In order to assess the validity of this model, the response of iron overloaded HepG2 cells upon iron chelator treatment were investigated with regards labile and total iron within cells, ROS generation and TfR1 expression. A reduction in iron concentrations were observed, which is consistent with earlier reports^39,44^. Further, generation of intracellular ROS was also reduced with chelator treatment suggesting a protective action which is in accordance with Huang et al*.*’s report. Moreover, TfR1 expression was also modulated by changes in the cellular iron status^39^. TfR1 expression is regulated by iron regulatory proteins by binding to iron-responsive elements in the 3’-untranslated region of TfR1 mRNA transcripts such that there is decreased expression under iron overload conditions^68^. As such, TfR1 expression was reduced upon iron loading followed by a subsequent increase with chelator treatment. Such intracellular changes are observed elsewhere with both TfR1 and intracellular ferritin^42^. It is interesting to note that TfR1 expression were significantly higher for DFO and DFX treated cells when compared to non-iron overload control cells (*p* < 0.0001 for both chelators), as observed by Chenoufi et al.^42^ One limitation of here is that the experimental concentration of chelators are not optimized for their iron chelation denticity (DFO = hexadentate, DFX = tridentate, DFP = bidentate) which can affect the free iron present, thus the efficacy. Consistent expression of ASGPR and TfR1 supports that the iron treatment does not impact cellular physiology. Irrespective of that the experiment with iron chelators demonstrates one of many applications for a well-defined and reproducible in vitro iron overload model.

Taken together, we document a process to develop an iron overload HepG2 model for consistency and reproducibility within the field. There is a plethora of investigations into the consequences of hepatic iron overload on systemic dysregulation, however, existing in vitro iron overload models are undermined by the lack of consensus with regards to iron source, iron loading concentrations and iron treatment durations. We showed that HepG2 cells treated with 50 μM iron-enriched media every 24 h for 48 h, followed by maintenance in fresh media for 24 h establishes an adequate iron overload model, as measured by changes in labile iron concentrations, total iron concentrations, ROS generation, indices of cytotoxicity, and protein expression. It is imperative to highlight the rigor for reproducibility in in vitro studies since these are often the frontline validation experiments that impact translation into in vivo investigations. Further, we believe this model is highly useful to study the adverse effects of liver parenchymal iron overload resulting in hereditary hemochromatosis or transfusion dependent thalassemia using an external non-physiological source of iron in vitro*.* However, further optimizations might be needed to tune this model to suit clinical relevance as the source of iron, loading kinetics of iron, tolerance, and biological behaviour of the cell line are detrimental factors.

## Conclusions

We reported the development of an iron overload HepG2 cell model for the primary purpose of demonstrating the importance of being diligent about the iron source, iron treatment conditions and duration of the treatment. Iron loading cells with 50 μM of iron every 24 h for 2 days, followed by maintenance for an additional 24 h of in fresh media. In doing so, we demonstrated that only 50 μM iron is required to result in increased iron-dependent measured outcomes—ROS generation, labile iron and total iron concentrations. Moreover, 50 μM of iron was sufficiently mild enough to maintain cellular functions such that it closely resembled control HepG2 cells. A robust protocol to establish iron overloading in HepG2 cells is of great importance as it highlights the current lack of consensus within the literature which translates into a lack of reproducibility with regards to iron-dependent measured outcomes and cellular functions. This process is adaptable to any other cell type.

### Supplementary Information

Supplementary Information.

## Data Availability

All data is available in the main text or the supplementary materials.
