# Supplementary material for: Development of an iron overload HepG2 cell model using ferrous ammonium citrate
Source: Sci Rep. 2023 Dec 8;13:21915. doi: 10.1038/s41598-023-49072-7 (PMC10713717; doi:10.1038/s41598-023-49072-7)
Supplement: Supplementary file 1 — Supplementary Information. [file 41598_2023_49072_MOESM1_ESM.docx]

**Development of an iron overload HepG2 cell model using ferrous ammonium citrate**

Usama Abbasi,^1,2,#^ Srinivas Abbina,^1,2,#^ Arshdeep Gill^1,2^, Jayachandran N. Kizhakkedathu^1,2,3,4,*^

^1^Department of Pathology and Laboratory Medicine, The University of British Columbia, Vancouver, BC, Canada.

^2^Centre for Blood Research, Life Sciences Institute, The University of British Columbia, Vancouver, BC, Canada.

^3^Department of Chemistry, The University of British Columbia, Vancouver, BC, Canada.

^4^The School of Biomedical Engineering, The University of British Columbia, Vancouver, BC, Canada.

^#^Equally contributed

^*^Address to which correspondence to be addressed.

**Supplementary Table and Figures:**

**Supplementary Table S1.** Literature reported iron overload models with different treatment regimes.

| Iron source | Iron concentration | Time of incubation | Cell lines | Outcome |
| --- | --- | --- | --- | --- |
| Ferric ammonium  citrate (FAC) | 100 or 200 μg/mL | 24 hr | HepG2 | No statistical difference in ferritin-L levels from 100 to 200 μg/mL^26^ |
| Fe-NTA | 5, 20, and 50 μM | 5 days | HepG2 | A slight difference in intra cellular iron was observed among the tested concentrations; however, it is not in linear relationship with iron loading^27^ |
| Fe-NTA | 30, 100, 300, 500, and 1000 μM | 6 days | HepG2 | 300 μM was chosen for mitochondrial ROS production considering the cytotoxicity^28^ |
| FAC | 720 μg/mL | 3-7 days | HepG2 | Consistent iron loading was observed and was chosen for lipid peroxidation assays^31^ |
| Ferrous sulfate | 1000 μM | 72 hr | HepG2 | Desired iron overload was achieved ^32^ |
| Ferrous sulfate | 10, 100, and 1000 μM | 3 days | HepG 2.2.15 | No statical difference was found among the treatments^33^ |
| Fe-NTA | 100 μM | 24 hr | HepG2  /HFE | Intracellular iron loading was observed^35^ |
| FAC or diferric transferrin | 66 μM | 7 or 10 days | HepG2, Hep3B | Maximum iron content was observed after 10 days^36^ |
| Ferrous sulfate heptahydrate or ferric sulfate.5H_2_O | 0.5 mM | 24 h | HepG2 | Intracellular iron was observed and used for chelating studies^38^ |
| Ferric citrate | 10 mM | 20 h | HepG2 | Iron chelator L1 was used in these studies to prevent DNA damage^39^ |
| Ferric saccharate, ferric gluconate, and ferric dextran | 25, 75, and 150 μM | 24 h | HepG2 | Determination of cellular iron levels^49^ |
| Iron citrate (Fe^3+^), iron sulfate (Fe^2+^), and iron chloride (Fe^3+^) | 10 mM | 4 h | HepG2 | Proliferation, toxicity, and hepcidin expression were measured^52^ |
| FAC | 5 mM | 12–72 h | HH4 | Intracellular ROS studies were performed^75^ |
| FAC | 200 or 300 µM | 24 or 48 h | Chondro-cytes | ROS studies were performed^76^ |


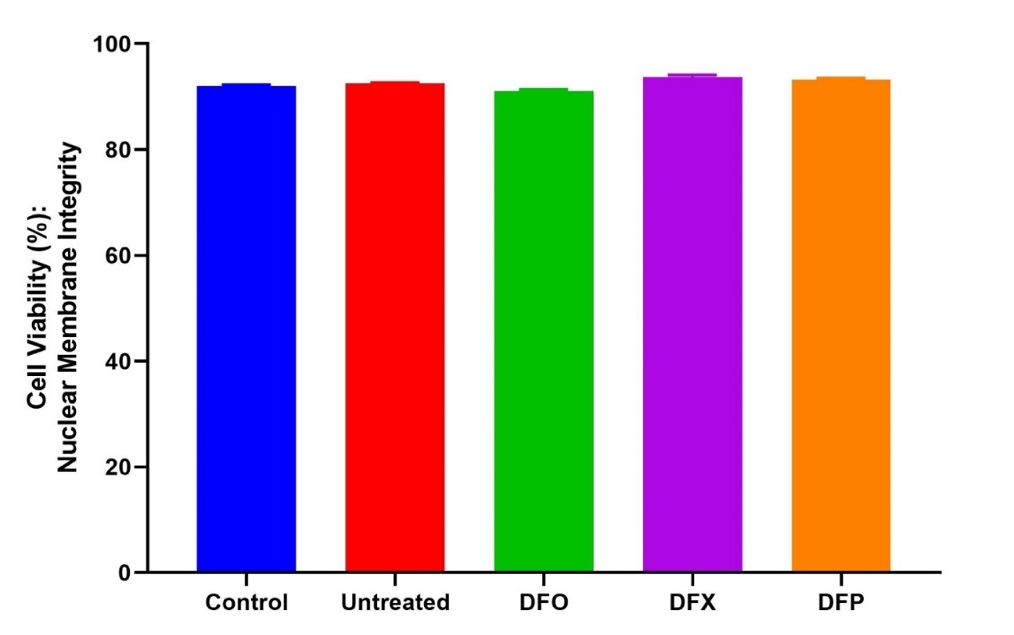


**Supplementary Figure S1**. Cell viability was assessed by MTT assay after iron overloaded HepG2 cells treated with small molecular iron chelators. Cells were seeded in a 48 well plate at a density of 50,000 cells per well. The MTT (3-(4,5-dimethylthiazolyl-2)-2,5-diphenyltetrazolium bromide) assay (ATCC 20-1010K) was performed according to manufacturer’s protocol to investigate changes in metabolic activity. Absorbance was measured at 570 nm on SpectraMax 190 Microplate Reader from Molecular Devices. Cell viability was determined; (mean_570 nm_ treated cells / mean_570 nm_ untreated cells) x 100%. Error bars show standard deviations for N = 3 independent replicates.


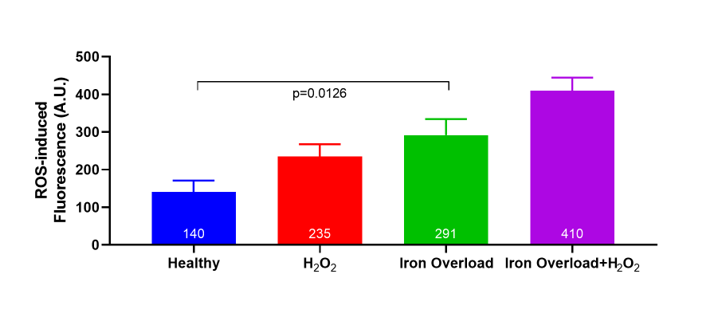


**Supplementary Figure S2**. Cellular ROS generation measured using a DCFDA / H2DCFDA kit (Abcam 113851) on Beckman Coulters CytoFLEX Flow Cytometer. One-way ANOVA with Dunnett’s correction method was used to compare among the treatments. Error bars show standard deviations for N = 3 independent replicates. Statistical analysis were performed using GraphPad Prism. **** represent p < 0.0001, *** represents p < 0.0010, ** represents p < 0.0100 and * represents p < 0.05.


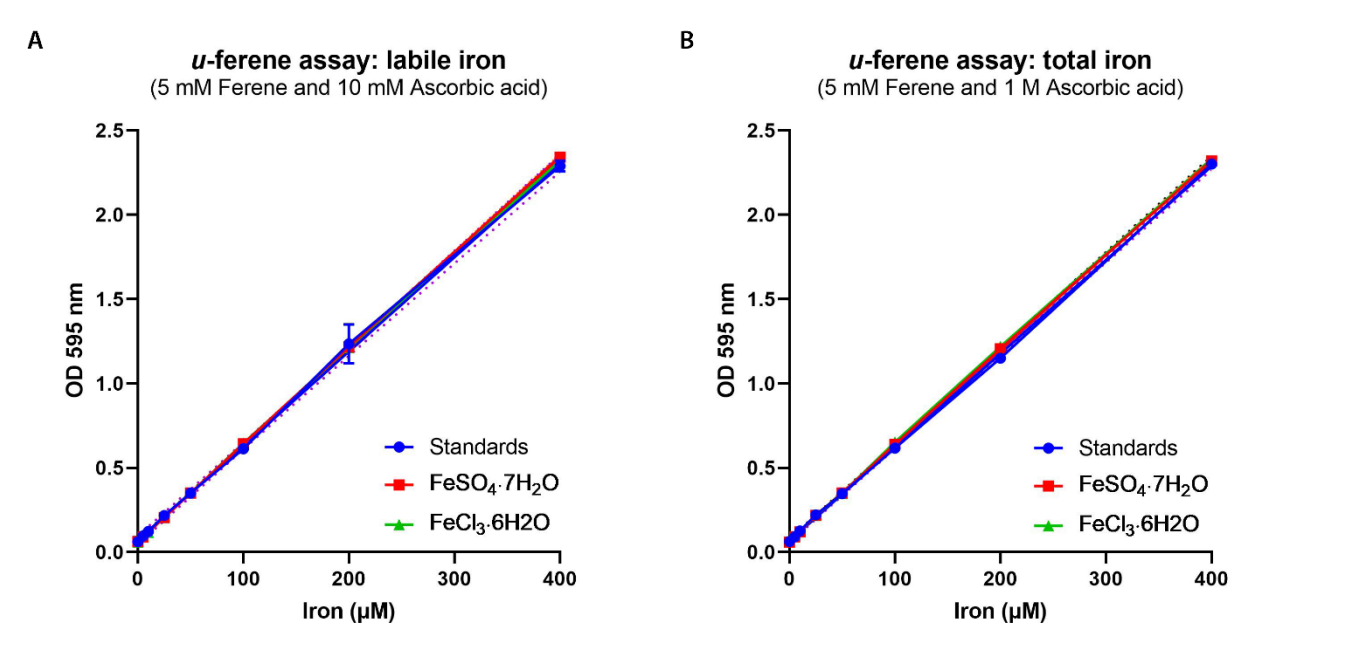

**Supplementary Figure S3.** Labile iron and total iron measurements were performed using u-ferene assay. HepG2 cells were overloaded with iron using FeCl_3_ and FeSO_4_ (Abbasi *et al.,* *Sci Rep*. **2021**, *11*, 6008).


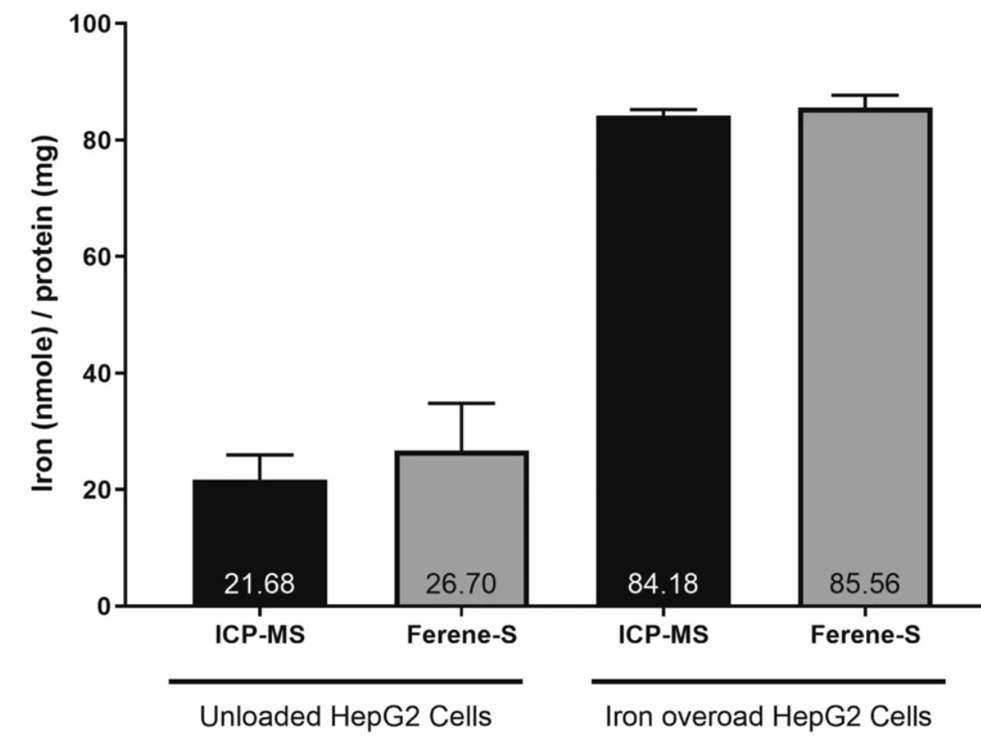


**Supplementary Figure S4**. Measurement of iron were performed using inductively coupled plasma mass spectrometry and the u-ferene assay in unloaded and iron overloaded HepG2 cells. We observed similar iron concentrations in both methods.

(**A**)





(**B**)





(**C**)





(**D**)


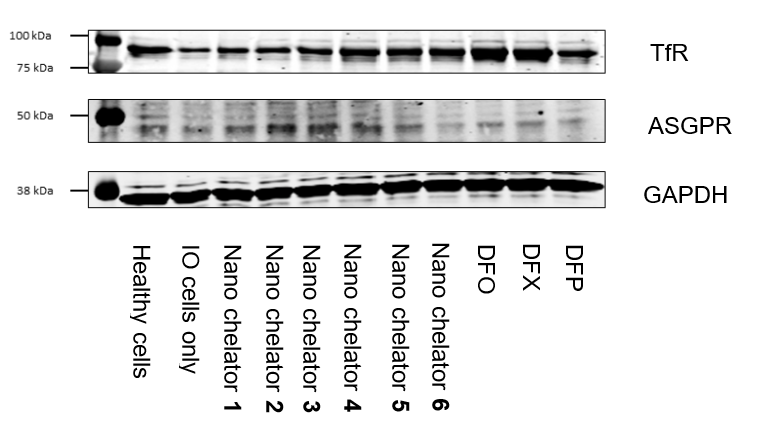


**Supplementary Figure S5**. Expression of key cellular trafficking proteins, (A) transferrin receptor (TfR1, 100 kDa), (B) asialoglycoprotein receptor (ASGPR, 42 kDa), and (C) house keeping protein, glyceraldehyde 3-phosphate dehydrogenase (GAPDH, 37 kDa) under iron overload (IO) conditions was assessed through western blotting. (D) All cropped versions are provided. Iron overloaded cells were treated with different newly developed nano chelators for iron chelation (in preparation) as well as FDA approved small molecular iron chelators. The protein expression is consistent through the treatment, or it is recovered.

**Supplementary Figure S6:** Surface levels of GAPDH and Ferroportin were measured via flow cytometry in iron overloaded HepG2 cells. The results are expressed as a calculated staining index which was calculated from the median fluorescence intensity of both the positive and negative controls. Data is representative of N=3 biological replicates. Error bars shown represent standard deviation. Refer experimental section for details.

**Supplementary Figure S7.** Primary antibodies were titrated on flow cytometer to find optimal concentration needed in Iron overload study. The Δ Staining Index was calculated as follows : $\Delta=\frac{\left( Positive MFI Signal \right)-(Negative MFI Signal)}{2*(Negative Signal rSD)}$ where MFI is Median Fluorescence Intensity. The optimal concentration was deemed to be 5% for both antibodies as that is when the titration curves flatten out thus indicating that the antibody binding has reached equilibrium. These concentrations represent final dilution in cell mixture. A detailed flow protocol on antibodies was found in methods section. Error bars represent standard deviation for N=3 biological replicates.
